# Development and application of the Chinese version of the adult strabismus quality of life questionnaire (AS-20): a cross-sectional study

**DOI:** 10.1186/1477-7525-11-180

**Published:** 2013-10-29

**Authors:** Zong-Hua Wang, Wei Bian, Hui Ren, Rosemary Frey, Ling-Fei Tang, Xian-Yuan Wang

**Affiliations:** 1 School of Nursing, Third Military Medical University, Postal address: Gaotanyan Street 30, Shapingba District, Chongqing 400038, China; 2 Southwest Eye Hospital, Third Military Medical University, Chongqing China; 3 School of Nursing, University of Auckland, Auckland, New Zealand; 4 Department of Psychology, Wayne State University, Detroit, Michigan, USA

**Keywords:** Strabismus, Quality of life, Questionnaires, Chinese

## Abstract

**Background:**

Patients with strabismus experience visual dysfunction, self-image disorders, low self-esteem, and social and emotional barriers, which adversely influence their health-related quality of life (HRQoL). Currently no strabismus-specific questionnaire is available in China to identify patients’ quality of life and to evaluate the effectiveness of strabismus treatment. The aims of the present study were to validate the Chinese-language version of the Adult Strabismus Quality of Life Questionnaire (AS-20) and to evaluate the impacts of strabismus on the quality of life among Chinese strabismus patients.

**Methods:**

Two hundred and fifty-five Chinese adults with strabismus, one hundred visually normal adults and one hundred patients with other eye diseases completed the Chinese version of AS-20. Psychometric properties of the Chinese AS-20 were examined by Cronbach’s α coefficient, test-retest and split-half reliability, and construct and criterion-related validity. Independent-samples t test and one-way ANOVA analyses were conducted to explore the impact of demographic factors and clinical characteristics on HRQoL in Chinese strabismic adults.

**Results:**

The final AS-20 in Chinese (AS-C) included 18 items and two subscales: psychosocial (12 items) and function (6 items). The Cronbach’s α was 0.908 for overall scale, with 0.913 and 0.808 for 'psychosocial’ and 'function’ subscales respectively, indicating high internal consistency reliability. The mean of the overall AS-C score among strabismus patients was 62.80 ± 18.94, significantly lower than that in visually normal adults (t = -18.693, P < 0.001), and in patients with other eye diseases (t = -5.512, P < 0.001).

**Conclusions:**

The AS-C is a culturally appropriate tool to evaluate the HRQoL in Chinese strabismus adults. The psychosocial health well-being and overall quality of life in strabismic patients should receive greater emphasis.

## Background

Strabismus has been reported to cause visual dysfunction, self-image disorders, low self-esteem, and social and emotional barriers, which adversely influence patients’ health-related quality of life (HRQoL) [1,2]. Individuals with strabismus fail to achieve proper binocular vision. They are unable to focus each eye on the same point in space due to a lack of coordination of extra-ocular muscles. Additionally, the appearance of misalignment results in social prejudice - associating strabismus with personality defects and below average intelligence [1,3]. Social prejudice relating to strabismus extends beyond social relationships. Adults with strabismus are likely to develop mannerisms to camouflage their dysfunction and to avoid eye contact during social interactions [3,4]. They also claim that their strabismus negatively affects job security and career opportunities [1].

A number of generic or strabismus-specific HRQoL instruments have been developed and applied. Such measures help to provide evidence-based healthcare, and help to evaluate the effectiveness of interventions. These scales include vision-specific ones like the National Eye Institute Visual Function Questionnaire (NEI-VFQ: 51- and 25-item questionnaires) [5,6] and the Impact of Visual Impairment (IVI) [7], and strabismus-specific ones including the Amblyopia and Strabismus Questionnaire (A&SQ) [8,9] and the Adult Strabismus Questionnaire (AS-20) [10].

The adult strabismus questionnaire (AS-20) contains 20 quality-of-life (QoL) related items generated from strabismus patients’ interviews. The AS-20 reported good internal consistency (Cronbach’s α = 0.94) [10] and a strong correlation with a widely used self-report QoL questionnaire: Derriford Appearance Scale 59 (DAS59) [11]. In addition, compared to NEI-VFQ-25, it demonstrated greater sensitivity [12] and better test-retest reliability [13] in strabismus adults. Hatt and colleagues [14,15] applied the AS-20 to evaluate HRQoL at the time points of six months and one year following successful strabismus surgery, and found that the AS-20 was able to better detect the improvement on HRQoL after successful strabismus surgery than NEI-VFQ-25.

The AS-20 has many advantages over other HRQoL instruments. First, the items were developed through strabismus patients’ self-reported concerns, while many other scales only considered the perspectives of physicians or researchers [16]. Second, items that simply describe presence or severity of symptoms are not included. The exclusion of both types of questions would improve the response accuracy and sensitivity. Third, items in the AS-20 are worded in simple sentences, allowing for ease of understanding by patients. Finally, the abbreviated length of the AS-20 allows easy and quick administration in clinical settings and communities.

The accumulated research evidence has led to growing concerns about the quality of life among strabismus patients in China. Nevertheless, neither general instruments like World Health Organization Quality of Life (WHOQOL) nor the ocular-specific scale like Chinese NEI-VFQ-25 [17] can specifically evaluate the HRQoL among strabismus patients. Therefore the purposes of this study were twofold: to develop and validate the Chinese version AS-20 among Chinese strabismus adults, and to evaluate the impact of strabismus on the QoL among Chinese strabismus patients.

## Methods

### Patients

Two hundred fifty-five Chinese strabismic adults were recruited from the outpatient clinic of Southwest Eye Hospital (Chongqing, China). A subsample of them (n = 60) were selected randomly to complete the Chinese version NEI-VFQ-25. Inclusion criteria were as follows: (1) aged 18 years or older, (2) understand and read Mandarin, (3) no history of emotional disorders including anxiety and depression, (4) no history of any eye-related surgery before participation, (5) no other facial or ocular abnormalities or acute eye diseases, (6) visual acuity in the better-seeing eye ≥ 20/50, and (7) the angle of deviation by prism was equal to or greater than 15 PD. Additionally, from the same outpatient clinic, one hundred visually normal adults and one hundred patients with other eye diseases (both groups are orthophoric adults) were recruited to complete the Chinese version AS-20. In all visually normal subjects, stereo acuity was examined by Titmus test (median, 40 seconds of arc); and visual acuity in better-seeing eye was at least 20/25 (median, 20/20 in each eye). In patients with other eye diseases, diagnoses were: retinal detachment (n = 23), vitreous haemorrhage (n = 18), cataract (n = 30), glaucoma (n = 19), and ocular trauma (n = 10); and visual acuity ranged from 20/20 to 20/40 (median, 20/30) for the better eye and from 20/20 to 20/80 (median, 20/40) for the worse eye. General information including demographic data about age, gender, education, and socioeconomic status, and eye-related information about types of strabismus and diplopia was recorded. No statistically significant differences were found between the study groups (strabismus patients, visually normal adults, and patients with other eye diseases) based on age, gender, marital status, and educational level.

### Instruments

The original AS-20 is organized into two subscales 'psychosocial’ and 'function’. The former subscale covers items associated with psychosocial functioning and self-awareness, while the latter one measures physical and emotional functions. Each subscale consists of 10 items rated on a 5-point Likert-type scale: never (score 100), rarely (75), sometimes (50), often (25), and always (0). The composite score is derived from the mean of all the questions answered. Median scores range from 0 to 100, with higher scores indicating better quality of life. The Cronbach’s α was 0.94 for the overall scale, with 0.95 for the psychosocial subscale and 0.94 for the function subscale [10].

The NEI-VFQ-25 is one of the most widely used instruments in ophthalmology research for assessing both self-reported visual functions and vision-related quality of life among people with eye diseases. It consists of 25 questions in 12 subscales: general health, general vision, ocular pain, near activities, distance activities, vision-specific social function, vision-specific mental health, vision-specific role difficulties, vision-specific dependency, driving, colour vision, and peripheral vision. Each item was scored on a five point scale from 0 (lowest QoL) to 100 (best QoL). The total score is calculated by averaging all of the items’ responses except for the 'general-health’ question, which is treated as a stand-alone item. The reliability of Cronbach’s α ranged from 0.71 to 0.85 [11]. Wang and associates translated and validated the scale in Chinese. The reliability of the Chinese version of NEI-VFQ-25 was satisfactory, with Cronbach’s α ranging from 0.73 to 0.87 [17].

### Translation and adaptation

The Chinese version of AS-20 was developed following standardized forward-backward translation procedure [18]. An independent forward translation was produced by two postgraduate students whose first language is Mandarin, and who are also fluent in English. Then the two postgraduates met with an expert panel consisting of one ophthalmologic doctor, two ophthalmologic nurses, one charge nurse and one psychological professor to discuss the forward translation in terms of content, semantic, technical equivalence, and cross-cultural adaptation, to finally produce the Chinese copy of AS-20. The translated copy was then separately sent to two healthcare-related bilingual speakers who performed blind backward translation; and their translations were compared with the original English version. Based on the expert panel’s suggestions, the reconciled forward translation was properly revised by researchers to establish the final version. The final version of the Chinese AS-20 was piloted with five strabismus patients and two ophthalmologists to identify possible misunderstanding of the items. Revisions were made until an acceptable Chinese translation was agreed upon.

### Data collection

All data in this study were collected among participants without having taken any strabismus-related surgery before. Adult strabismus patients who met the inclusion criteria were invited on the first day of visiting the outpatient clinics. After receiving verbal and written instructions, each participant was left alone in the waiting room to complete the Chinese version AS-20. When patients came to the clinics again for further examinations, sixty out of the total sample were chosen randomly to complete the Chinese version NEI-VFQ-25 on the following day, and fifty of the total participants were chosen randomly to complete the Chinese AS-20 again to establish test-retest reliability on the seventh day.

### Data analysis

Data analyses were carried out using SPSS (Version 20.0). All the analyses were interpreted two-sided, being fixed at a 5% level of significance. According to age, gender, education level, and clinical features, the differences in quality of life among strabismus patients were compared using one-way ANOVA analyses or independent-samples t tests. Levene’s Test was conducted for assessing the equality of variances in preparation for independent-samples t test. In terms of psychometric properties, content validity, construct validity, criterion-related validity, internal consistency, test-retest reliability, and split-half reliability were assessed.

Discriminant validity was established in this study by conducting an extreme group analysis which involved assessing the significance of the difference between the upper 27% and lower 27% groups’ item scores. Additionally, Spearman’s coefficient was used to determine the relationship between the score of each item and the composite score. The coefficient was evaluated according to the following levels: less than 0.30, weak correlations and little clinical applicability, even when statistically significant; between 0.30 and 0.50, moderate correlations and above 0.50, strong correlations. Any item with coefficient under 0.40 would be deleted [19,20]. Criterion-related validity was evaluated by determining the Spearman’s rank order correlation coefficient between the AS-20 and the NEI-VFQ-25.

## Results

### Demographic data and clinical characteristics

A convenience sample of 268 strabismus patients was invited to take part in this study. Four questionnaires were not returned, and nine were incorrectly completed. Thus 255 (95.15%) valid questionnaires were available for statistical analysis. The average age of all strabismus patients was 27.10 ± 9.15 years old. Thirty-six (14.1%) of the participants never received any family support, and 164 (64.3%) self-reported no health insurance support; One hundred and thirty-five (52.9%) of the patients had exotropic strabismus, and 88 (34.5%) suffered from double vision (Table 1). The diagnoses for the 88 strabismus patients with diplopia were: congenital/infantile strabismus (n = 19), intermittent exotropia (n = 25), neurogenic strabismus (n = 16), sensory (n = 10), accommodative esotropia (n = 7), and acute comitant esotropia (n = 11).

**Table 1:** Demographic and clinical characteristics of the adult strabismus (N = 255)

	**Strabismus adults n (%)**	**AS-C score (Mean ± SD)**		
		**Total scale**	**Psychosocial**	**Function**
**Gender**				
Male	134 (52.55)	66.38 ± 19.61^**^	61.68 ± 22.65^**^	75.78 ± 20.33
Female	121 (47.45)	58.83 ± 17.40	51.05 ± 20.29	74.38 ± 21.04
**Family support**				
Never	36 (14.12)	50.93 ± 19.58^*^	45.49 ± 23.37^*^	61.81 ± 22.92^*^
Sometimes	99 (38.82)	64.79 ± 17.54	58.78 ± 21.27	76.81 ± 18.71
Always	120 (47.06)	64.71 ± 18.70	58.21 ± 21.71	77.71 ± 20.09
**Education level**				
Primary school and lower	91 (35.69)	61.20 ± 18.05	55.24 ± 21.56	73.12 ± 20.56
High school	94 (36.86)	61.11 ± 18.77	55.60 ± 22.42	72.12 ± 19.70
University degree and higher	70 (27.45)	67.14 ± 19.86	59.85 ± 22.59	81.73 ± 20.80^*^
**Socioeconomic status**				
Urban	101 (39.61)	61.01 ± 20.16	54.70 ± 23.46	73.64 ± 21.64
Rural	154 (60.39)	63.96 ± 18.07	57.91 ± 21.26	76.08 ± 19.97
**With double vision/Diplopia**				
Yes	88 (34.51)	57.38 ± 18.59^**^	53.25 ± 21.50	65.62 ± 21.81^**^
No	167 (65.49)	65.65 ± 18.55	58.42 ± 22.37	80.11 ± 18.15
**Types of strabismus**				
Esotropia	120 (47.06)	59.55 ± 18.86^*^	52.55 ± 21.82^**^	73.54 ± 22.01
Exotropia	135 (52.94)	65.68 ± 18.61	60.72 ± 21.91	76.51 ± 19.32
**Deviation size**				
≤ 25 PD	53 (20.78)	64.31 ± 20.43	58.84 ± 24.47	75.24 ± 19.67
> 25 PD	202 (79.22)	62.40 ± 18.56	56.06 ± 21.55	75.08 ± 20.93
**Health insurance support**				
Yes	91 (35.69)	63.29 ± 19.81	57.11 ± 22.88	75.66 ± 21.07
No	164 (64.31)	61.90 ± 17.33	55.79 ± 20.92	74.13 ± 19.92

Education level and family support utilized one-way ANOVA analyses. Independent-samples t tests were conducted with the other variables. Levene’s Test was conducted to verify the equality of variances.

> 25 PD, ≤ 25 PD: Deviation size for esotropia and exotropia.

^**^P < 0.01, two sides, ^*^P < 0.05, two sides.

### Content validity

Content validity was assessed by the expert panel who scored the relevance of each item on a 4-point scale of 1 = not relevant, 2 = somewhat relevant, 3 = quite relevant, and 4 = very relevant according to instrument’s purpose and connotation. Based upon their answers, a content validity index (CVI) [21], the percentage of items received a rating of 3 or 4, was calculated. The items that had CVI over 0.80 [22] remained. For the Chinese version of the AS-20, the average CVI was 0.93 indicating satisfactory content validity, and no item was deleted. According to the experts’ feedback, item 18 “I worry about my eyes” was reworded to “I worry about my eyes getting worse” to improve cultural understanding.

### Construct validity

The Kaiser-Meyer-Olkin (KMO) for the 20-item Chinese AS scale was 0.924 and the Bartlett’s test for sphericity was significant (P < 0.001) [20], suggesting the Chinese AS-20 was suitable for principal component analysis (PCA). After conducting PCA with Varimax rotation, unlike the original scale, the Chinese AS-20 extracted three factors with eigenvalues greater than 1.0, explaining 56.39% of the total variance. Specifically, the first item “I worry about what people will think about my eyes” showed equivalent correlation within factor 1 and factor 3 (r = 0.573 vs. 0.539), while the fifteenth item “My eyes feel strained” showed similar correlations with factor 2 and factor 3 (r = 0.502 vs. 0.564). After discussion with two psychological experts, both items were deleted.

A second PCA with Varimax rotation was conducted. Two factors were extracted, explaining 52.57% of the total variance. Specifically, the first factor 'psychosocial’ accounted for 39.78% of the total variance, with the second factor 'function’ for 12.78%. Moreover, the results suggested that item 17 “I feel stressed because of my eyes”, item 18 “I worry about my eyes of getting worse” and item 19 “I can’t enjoy my hobbies because of my eyes” in the Chinese version AS-20 should be categorized into the factor “psychosocial”. Based on cultural considerations, the expert panel and all researchers agreed upon this new scale structure, since the words “stressed” “worry” “enjoy” were more associated with emotional feelings rather than functional abilities in Chinese culture.

Therefore all the following analyses in terms of reliability and validity were conducted on the basis of the new structure, using the remaining 18 items in two subscales. The first subscale 'psychosocial’ contained 12 items, while the second one 'function’ included six items. This scale was entitled the Adult Strabismus in Chinese (AS-C) for better illustration. Regarding the factor loading of the 18 items, moderate to strong loadings were found with one of the two factors (range from 0.421 to 0.658) (Table 2).

**Table 2:** Psychometric properties of the Chinese version AS-20 (AS-C)

**Subscale**	**Item number**	**Critical ratio (CR)**	**Factor loading**	**Test-retest reliability**	**Cronbach’s Alpha if item deleted**	**Split-half reliability**
**Psychosocial**						0.889
People are thinking about my eyes	2	15.518^**^	0.615	0.510^**^	0.901	
Feel uncomfortable	3	9.871^**^	0.532	0.339^*^	0.905	
Wonder what people are thinking	4	11.231^**^	0.536	0.587^**^	0.903	
Don’t give me opportunities	5	12.476^**^	0.421	0.357^*^	0.903	
Feel self-conscious	6	16.744^**^	0.655	0.720^**^	0.899	
Avoid looking at me	7	11.092^**^	0.449	0.562^**^	0.903	
Feel inferior	8	15.992^*^	0.644	0.697^**^	0.899	
React differently	9	11.039^**^	0.481	0.588^**^	0.902	
Hard to initiate contact	10	13.699^**^	0.535	0.516^**^	0.902	
Feel stressed	17	19.460^**^	0.658	0.392^*^	0.897	
Worry about my eyes	18	12.079^**^	0.422	0.446^**^	0.903	
Can’t enjoy my hobbies	19	14.993^**^	0.476	0.414^**^	0.901	
**Function**						0.803
Cover or close one eye	11	7.148^**^	0.425	0.648^**^	0.907	
Avoid reading	12	6.969^**^	0.483	0.388^*^	0.907	
Stop doing things	13	6.097^**^	0.469	0.673^**^	0.908	
Depth perception	14	9.262^**^	0.488	0.575^**^	0.905	
Have problems reading	16	7.103^**^	0.614	0.731^**^	0.907	
Frequent breaks	20	10.326^**^	0.561	0.548^**^	0.905	

^**^P < 0.01, two sides, ^*^P < 0.05, two sides.

### Convergent/discriminant validity

All items in AS-C produced significant differences between the upper 27% group and the lower 27% group (CR 6.097 ~ 19.460, P < 0.01) (Table 2). In addition, the strength of relationship between scores of each subscale and the total score supported the convergent validity. Each item revealed statistically adequate correlations with one of two subscales as well as the total scale, with Spearman coefficients ranging from 0.442 ~ 0.801 (P < 0.01) (Table 3). At the same time, the scores of each subscale showed a strong correlation with the overall score (P < 0.01) (Table 3), and relatively weak correlation between subscales each other (P < 0.01) (Table 3).

**Table 3:** Correlation between scores of each subscale and the total score

**Items**	**Psychosocial**	**Function**	**Overall**
People are thinking about my eyes	0.780^**^	0.314^**^	0.735^**^
Feel uncomfortable	0.662^**^	0.132^**^	0.566^**^
Wonder what people are thinking	0.699^**^	0.236^**^	0.628^**^
Don’t give me opportunities	0.655^**^	0.264^**^	0.622^**^
Feel self-conscious	0.811^**^	0.272^**^	0.739^**^
Avoid looking at me	0.653^**^	0.305^**^	0.620^**^
Feel inferior	0.791^**^	0.318^**^	0.742^**^
React differently	0.674^**^	0.293^**^	0.641^**^
Hard to initiate contact	0.706^**^	0.256^**^	0.650^**^
Feel stressed	0.791^**^	0.483^**^	0.812^**^
Worry about my eyes	0.613^**^	0.432^**^	0.643^**^
Can’t enjoy my hobbies	0.687^**^	0.391^**^	0.700^**^
Cover or close one eye	0.284^**^	0.676^**^	0.452^**^
Avoid reading	0.306^**^	0.625^**^	0.462^**^
Stop doing things	0.223^**^	0.705^**^	0.413^**^
Depth perception	0.385^**^	0.685^**^	0.533^**^
Have problems reading	0.275^**^	0.726^**^	0.470^**^
Frequent breaks	0.389^**^	0.751^**^	0.560^**^
**Psychosocial**	1	0.452^**^	0.949^**^
**Function**	0.452^**^	1	0.673^**^
**Overall**	0.949^**^	0.673^**^	1

Spearman rho coefficient. ^**^P < 0.01, two sides.

### Criterion-related validity

Both the overall AS-C (r = 0.314, P < 0.05) and the 'function’ subscale (r = 0.476, P < 0.01) showed moderate positive correlations with the NEI-VFQ-25. No significant correlation was found between the 'psychosocial’ subscale and the NEI-VFQ-25 (r = 0.195, P > 0.05).

### Reliability

The Cronbach’s α for overall AS-C was 0.908 indicating high internal consistency and homogeneity of the scale, with 0.913 and 0.808 for the 'psychosocial’ and 'function’ subscale, respectively. For the test-retest reliability, the Spearman’s coefficient ranged from 0.339 to 0.731 for each item and 0.906 for the overall score. The split-half reliability was 0.735, with 0.889 for the 'psychosocial’ subscale and 0.803 for the 'function’ subscale.

### The relationship of quality of life with demographic factors and clinical features

Women reported a significantly lower score for the overall scale (t = 3.239, P = 0.001) (Table 1) and for the psychosocial subscale (t = 3.932, P < 0.001) (Table 1) in comparison to men. Strabismic patients with university degree and higher had a significantly better function-related quality of life (P < 0.05) (Table 1). Compared to those who 'sometimes’ and 'always’ received family support, strabismic patients who reported never having family support recorded the lowest quality of life scores in overall AS-C score, psychosocial subscale, and function subscale (all P < 0.05) (Table 1). The total AS-C score for exotropic patients was significantly higher than that for esotropic patients (t = 2.611, P = 0.010) (Table 1), as well as for their score on psychosocial subscale (t = 2.812, P = 0.005) (Table 1). Strabismus patients with diplopia had significantly lower scores than those without diplopia in terms of overall scale (t = 3.384, P = 0.001) (Table 1) and the function subscale (t = 5.644, P < 0.001) (Table 1).

### Comparisons of AS-20 median scores

The mean of overall AS-C score among strabismus patients was 62.80 ± 18.94, significantly lower than that in visually normal group (median, 91.52; t = -18.693, P < 0.001) and other eye diseases adults (75.28; t = -5.512, P < 0.001). The median scores for the psychosocial subscale, were significantly lower in strabismus patients compared with visually normal adults (56.64 versus 94.70, t = -22.987, P < 0.001) and patients with other eye diseases (77.50, t = -7.833, P < 0.001). The median scores for the function subscale were again significantly lower in strabismus patients compared with visually normal adults (median, 75.11 versus 88.33, t = -7.257, P < 0.001), yet no significant difference has been demonstrated with other eye diseases in adults (versus 73.05, t = 0.864, P > 0.05). Further comparisons between the average scores for the two subscales indicated that the mean score for the psychosocial subscale was significantly lower than the mean score for the function subscale (56.64 versus 75.11, t = -9.742, P < 0.001).

## Discussion

In the present study, the original AS-20 scale was translated and adapted to the Chinese culture. The findings of this study revealed that the AS-C had excellent psychometric properties and provided equivalent evaluations to the original English version. Above all, the instrument is culturally appropriate for evaluating the health-related quality of life among strabismus adults in China.

Psychometric results suggested that the AS-C was a reliable, discriminative and stable instrument for evaluating health-related quality of life in Chinese strabismus adults. The whole scale, psychological and function subscale (Cronbach’s α = 0.908, 0.913 and 0.808, respectively) reported excellent internal consistency. These values were consistent with the original AS-20 (range, 0.94 to 0.95) [10]. Moreover, the test-retest reliability of the AS-C overall scale was 0.906, consistent with the result of 0.92 reported by Leske and associates [12], indicating the AS-C was reliable and stable for use across time. This study also measured the test-retest coefficient of each item, ranging from 0.339 to 0.731. This may imply that the AS-C could pick up patients’ emotional changes which vary over time. The split-half reliability of psychosocial and function subscale were 0.889 and 0.803 respectively, indicating good consistent reliability within each subscale. Additionally, the results of the extreme group comparisons suggested that the AS-C was able to discriminate among strabismus patients with different measured levels of quality of life. The AS-C can also distinguish the QoL among adult strabismus patients from that of visually normal adults and other eye diseases patients.

The results of content and construct validity supported that the Chinese version of AS-20 questionnaire was conceptually and culturally appropriate to evaluate the health-related quality of life among strabismus patients (CVI of 0.95). The convergent validity results indicated a strong correlation of the overall scale with each subscale, and a moderate correlation between subscales. The HRQoL among strabismus patients was confirmed to include two independent domains: psychosocial and function. Both of the aspects identified are homogeneous in measuring the concept of quality of life, but weakly related to one another.

Demographic factors and strabismus-related clinical features should be taken into consideration when evaluating quality of life of strabismus patients. Our results revealed that female gender, a low level of education, and lack of family support were barriers for strabismic adults to achieve a better quality of life. It should be pointed out that the patients in our cohort were very young (median age, 26.83 ± 9.03 years) compared to the previous studies (median age, 44 to 53 years) [11,12]. The reason we assume is that Chinese people have great concerns about self-esteem and “face”. The concept of 'face’ is very important in Chinese culture. “Face” can be defined as an individual’s contingent self-esteem [23]. It symbolises the individual’s social position or reputation in society, and represents acceptance and recognition in Chinese society [24]. Strabismus and misaligned appearance may result in 'losing face’ and feeling inferior. Patients therefore tend to visit doctors for strabismus treatment at an early age, allowing them regain self-confidence and promote self-development as early as possible. Further studies are needed to explore qualitative information and to examine whether self-esteem and confidence are improved after strabismus treatment.

Distinctions were also evident based on the type of strabismus. The negative impact of esotropia was more pronounced for the psychosocial aspect compared to exotropia, while the negative impact of diplopia was more evident for the functional aspect. Since the main problems with diplopia are judging where things are in space and depth perception, strabismus patients with double vision might report more function-related complaints than those without. Regarding exotropia and esotropia, Olitsky and associates [1] identified that strabismus faces were significantly related to negative social prejudice. The orthotropic face was more positively judged than the esotropic face with respect to sincerity, competency, intelligence, emotional stability, and communication skills, and the negative judgements on esotropia was more reported than that on exotropia. In summary, the results suggested that the psychosocial health well-being and overall quality of life in esotropic patients should receive more attention in clinical practices.

Significant decreases in quality of life among strabismus adults have been identified, especially on the aspect of psychosocial health wellbeing. The mean score of the overall AS-20 in Chinese strabismus patients was significantly lower than that in visually normal adults and patients with other eye diseases, indicating the negative influences of strabismus on Chinese patients’ quality of life. These results may suggest to clinical healthcare providers that more emphasis should be placed on the quality of life in Chinese strabismus patients, especially on psychosocial aspect. Interestingly, no significant difference was shown in function scale between patients with strabismus and those with other eye diseases while the original AS-20 did find a statistically significant difference [10]. The possible explanation is the low percentage of patients with diplopia in our study cohort (34.5%) compared to other studies (64% to 77%) [10-12,15]. As mentioned above, diplopia was more associated with function-related complaints.

As is the case with all research, some limitations to this study should be noted. First, we recruited a relatively small number of patients (n = 255) for exploring criterion-related validity and test-retest reliability. Nevertheless, it is noteworthy that we were still able to observe statistically significant correlations between the VFQ-25 and the AS-C, and between the scores at two different time points (the first and the seventh day). Another potential limitation is that the participants we recruited from outpatient clinics might be more concerned about their eyes’ functions and more unsatisfied with their misaligned appearance compared to patients who had strabismus but didn’t come to clinics; therefore our participants could show significantly lower scores on this scale. In addition, although the results did not indicate a significant difference in quality of life between patients receiving health insurance and those who were not, the amount and type of health insurance coverage may influence the level of quality of life in strabismus patients. Future research should explore possible differences based on the amount and type of health insurance. Finally, we have not performed item response theory analysis as the English version of the AS-20 has undergone such analysis; addition of such analyses will be helpful to remove inappropriate items and hence to improve the accuracy of the items. Nevertheless, the study results are still of relevance to healthcare professionals who work with those strabismus patients within clinical settings. In spite of the limitations, this study should be recognised for establishment and adaptation of the Chinese version of the AS-20, and identification of the psychometric properties of the AS-C. Furthermore, this study provides clinically-based evidence on the needs of strabismus patients to shape strabismus interventions to improve health-related quality of life [20].

## Conclusions

To our knowledge, this is the first study to develop and administer a strabismus-specific questionnaire to evaluate the impact of strabismus on the quality of life of patients in China. The findings of our study suggest good reliability, validity and stability of the Chinese version AS-20 questionnaire for clinical use. This instrument may support health care professionals to provide strabismus appropriate and friendly interventions. Further studies are suggested to examine whether and how HRQoL would change at 6 month or one year after strabismus surgery. Moreover, the cut-off points to distinguish normal quality of life and below among Chinese strabismus patients should be explored.

### Ethics consideration

Ethical approval was obtained from the human ethics committee of the First Affiliated Hospital of Third Military Medical University. Research information sheet was handed out and verbal instruction was given to patients before their participation. All participants gave their written informed consent. Whenever a subject decided to withdraw from the study, all data pertaining to the participant was destroyed. All study procedures were performed according to the Declaration of Helsinki.

## Abbreviations

HRQoL: Health-related quality of life; QoL: Quality of life; AS-20: The adult strabismus quality of life questionnaire; NEI-VFQ-25: The national eye institute visual function questionnaire-25; CVI: Content validity index; PCA: Principal component analysis; AS-C: The adult strabismus in Chinese.

## Competing interests

This study was funded by The First Affiliated Hospital of Third Military Medical University Nursing Innovation Research Grants. The sponsor or funding organization had no role in the design or conduct of this research, data analysis or manuscript preparation. No authors have any financial interest to disclose.

## Authors’ contributions

WX, RH and WZ developed the original concept for this study; WZ, BW and TL forward- and back- translated the AS-20 questionnaire; WZ and BW recruited patients, performed questionnaires, collected and analysed data; BW received funds for this study; WZ, RF and TL wrote and revised the manuscript; WX and RH managed and logistically support the study, coordinated between hospital and university, assisted to obtain the ethical approval. All authors read and approved the final manuscript for this study.
